# The Halo Vest Immobilization for Craniocervical Junction Tuberculosis: A Comparison of Treatment Options

**DOI:** 10.1111/os.70047

**Published:** 2025-05-01

**Authors:** Yunfeng Wu, Xiyou Yang, Long Yu, Ning Liu, Shangjie Yang, Xu Cui

**Affiliations:** ^1^ College of Medicine Southwest Jiaotong University Chengdu Sichuan Province China; ^2^ Department of Orthopedics, The Eighth Medical Centre Chinese PLA General Hospital Beijing China

**Keywords:** craniocervical junction, halo vest, nonsurgical management, occipitocervical fusion, tuberculosis

## Abstract

**Objective:**

The treatment of tuberculosis at the craniovertebral junction (CVJ) remains challenging, with significant debate surrounding therapeutic approaches. Halo vest (HV) therapy provides a non‐invasive immobilization alternative, while occipitocervical fusion (OCF) offers a surgical option. However, limited evidence exists comparing the efficacy of HV therapy with OCF for CVJ tuberculosis. This study aimed to evaluate the clinical outcomes and safety of HV immobilization in treating CVJ tuberculosis, compared with OCF.

**Methods:**

This retrospective cohort study was conducted from January 2012 to December 2022 and included 43 patients diagnosed with CVJ tuberculosis. Radiographic and treatment data were meticulously analyzed to compare outcomes between patients treated with HV immobilization (Group H, *n* = 22) and those undergoing OCF (Group O, *n* = 21). Interventions comprised at least 28 weeks of HV immobilization for Group H and OCF supplemented with postoperative external fixation for Group O. Outcomes were evaluated using the Visual Analog Scale (VAS‐neck), erythrocyte sedimentation rate (ESR), and radiographic stability (assessed via X‐ray and CT). Statistical analyses included the Student's *t*‐test (parametric data), Mann–Whitney *U* test (nonparametric data), and chi‐square test (categorical variables), with a significance level set at *p* < 0.05.

**Results:**

Over a 24‐month follow‐up, all patients exhibited successful healing of tuberculosis lesions. Group H demonstrated greater improvements in cervical flexion‐extension and rotation function compared with Group O. Both groups showed statistically significant decreases in Visual Analog Scale (VAS‐neck) and Neck Disability Index (NDI) scores, as well as in ESR and C‐reactive protein (CRP) values from pre‐surgery levels (*p* < 0.01). Notably, differences in VAS‐neck (1 month) and NDI (24 months) between the groups were statistically significant (*p* < 0.05), while no significant differences were observed in other follow‐up periods (*p* > 0.05). Additionally, there were no significant differences in ESR and CRP values at any time point between the treatment groups (*p* > 0.05).

**Conclusions:**

The study yielded satisfactory outcomes for all patients. Short‐term differences in pain relief did not significantly impact the healing of CVJ tuberculosis. Patients receiving HV treatment showed greater improvement in neck function compared with those undergoing occipitocervical fusion. Given the substantial costs and risks associated with open surgery, we advocate for conservative treatment utilizing HV.

## Graphical abstract

This study aimed to evaluate the clinical outcomes and safety of Halo vest (HV) immobilization in treating craniovertebral junction tuberculosis, compared with occipitocervical fusion. The results showed that patients receiving HV treatment had greater improvement in neck function compared with those undergoing occipitocervical fusion.

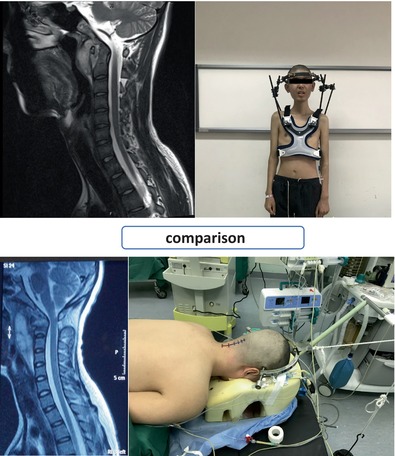

## 1 Introduction

Spinal tuberculosis (TB), occurring in < 1% of TB cases [1], remains one of the most severe forms of extrapulmonary infection, historically associated with 30%–50% mortality before multidrug therapy [2, 3, 4] Modern treatments have reduced mortality to < 1%; yet, its clinical impact persists, particularly in rare craniovertebral junction (CVJ) involvement (1%–5% of spinal TB) [5]. The CVJ—comprising the occipital bone, atlas, and axis—is the cervical spine's most mobile segment, where TB manifests as abscesses/granulomas with potential spinal cord or brainstem compression [6]. Despite the atlantoaxial canal's capacious anatomy allowing insidious progression (average symptom onset: 5 months) [7], advanced cases risk quadriplegia, bulbar dysfunction, or life‐threatening medullary compromise [8]. Common presentations include neck pain, spinal tenderness, and constitutional symptoms (fever, weight loss) [9, 10], while CVJ‐specific manifestations may involve dysphagia or dyspnea due to pharyngeal abscesses. Notably, some patients exhibit minimal neurological deficits despite extensive lesions, underscoring the disease's diagnostic challenge. In our cohort, severe cord compression signs (e.g., paraplegia) were absent, aligning with reports of delayed neurological deterioration in untreated cases.

The treatment of CVJ TB remains contentious. Geol et al. classified the disease into three stages based on radiological findings [11, 12]. Stage 1 patients, with mild symptoms, can be effectively managed with antitubercular chemotherapy, external orthoses, and prolonged rest [13]. Surgical intervention is recommended for patients with neurological deficits to ensure stability, with procedures such as posterior fusion or posterior fusion combined with anterior decompression often yielding favorable outcomes [14, 15]. However, most patients do not exhibit significant neurological compression, making the decision between conservative management and surgical treatment a challenging clinical dilemma [16, 17].

The halo vest (HV) treatment was first introduced by Perry and Nickel in 1959 [18]. It offers better stabilization than traditional rigid collar placement but less stabilization compared with operative fixation and fusion [19, 20]. As a non‐surgical intervention, it can be the primary treatment for many instabilities in the cervical spine, including some cases of CVJ TB.

The current treatment strategies for CVJ TB include non‐surgical approaches such as HV immobilization and surgical interventions like occipitocervical fusion (OCF). Although HV immobilization is minimally invasive, it is associated with prolonged immobilization discomfort and complications such as pin‐site infections [21]. Conversely, OCF effectively achieves spinal stability but carries risks of surgical complications and higher costs [22, 23]. Despite these options, consensus on the optimal treatment strategy remains lacking, particularly in resource‐limited settings. Additionally, due to the complex anatomy of the atlantoaxial region and the deep‐seated location of the CVJ, conventional anterior cervical submandibular approaches often fail to achieve extensive debridement of TB lesions (e.g., sequestra or caseous tissues) or reconstruction of posterior cervical stability. These limitations underscore the urgent need for comparative studies to guide clinical decision‐making. The objectives of this study are: (i) to compare the clinical efficacy of HV immobilization and OCF in CVJ TB; (ii) to evaluate complication rates and quality of life associated with each treatment modality; and (iii) to investigate whether non‐surgical management can serve as a viable alternative to surgery in selected cases.

## 2 Methods

This study is a retrospective single‐center cohort study. This study was approved by the Ethics Committee of The Eighth Medical Center of PLA General Hospital (No. 309202206171052).

### 2.1 Data Collection

This retrospective study was conducted at a supra‐regional TB center in northern China, enrolling patients diagnosed with CVJ TB between 2012 and 2022. Inclusion criteria comprised: (i) adults aged ≥ 14 years with complete clinical and radiographic data and a minimum 24‐month follow‐up; (ii) radiologically confirmed CVJ TB classified as Stage 2 or 3 according to Geol's criteria [11], defined as progressive bone destruction with bilateral facet involvement (Stage 2) or atlantoaxial instability/subluxation (Stage 3); and (iii) patients undergoing either halo‐vest (HV) immobilization (≥ 28 weeks) or OCF with postoperative external fixation. Exclusion criteria included severe neurological deficits, systemic comorbidities (e.g., active extrapulmonary TB), prior cervical surgery, or incomplete follow‐up.

### 2.2 Patient Characteristics

Based on these criteria, 43 patients were included in this study. Twenty‐eight were males and 15 were females; their ages ranged from 14 to 77 years. Of these, 21 were diagnosed with atlantoaxial TB (C1‐2), 15 with atlanto‐occipital TB (C0‐1), and 7 with infections involving the occipital, atlantoaxial, and axial spine (C0‐2). In the classification of progression patterns of CVJ TB, 27 patients were categorized in Stage 2, characterized by unilateral bony destruction of the atlantoaxial joint, with the contralateral joint remaining unaffected and no significant instability observed in the CVJ. 16 patients were categorized in Stage 3, exhibiting destruction of the contralateral joint, with bone destruction involving the contralateral atlantoaxial joint and other bones and joints in the region, yet without severe neurological impairment. The patients were followed up for 24 months. Before surgery, all patients received standard anti‐TB chemotherapy for a fortnight. The chemotherapy regimen consisted of isoniazid (INH) at a dose of 5 mg/kg once daily in a single dose; rifampicin (RFP) at a dose of 8 mg/kg once daily in a single dose 2 h before meals; pyrazinamide (PZA) at a dose of 20 mg/kg once daily in 3 divided doses; and ethambutol (EMB) at a dose of 12 mg/kg once daily in a single dose. At the same time, the liver‐protecting drug bicyclol was added, with a daily dose of 150 mg in 3 doses.

### 2.3 Surgical Procedures

All patients and their families were informed about the advantages and disadvantages of the two treatment options and chose accordingly. There were 22 patients treated with HV (Group H) and 21 patients with OCF (Group O).

#### 2.3.1 Group H

The HV was applied under local anesthesia in the supine position. The halo‐crown was adjusted with 4–6 pins depending on patient age and bone density. Half of the pins were placed in the frontal area and the other half over the parietal and occipital bones. After confirming the ability to swallow and talk, the halo‐crown and vest were connected with rods. The patient's condition, including breathing, swallowing, talking, and downward gaze, was monitored [24].

#### 2.3.2 Group O

In the prone position, all patients were secured in a Mayfield skull clamp under general anesthesia. Intraoperative traction was applied to all patients. A standard midline incision was made to expose the area from the external occipital protuberance to the spinous processes of the cervical spine. Polyaxial screws were used as either pedicle or lateral mass screws of the cervical spine as needed. The plate portion of the rod was slightly bent to fit the occipital contour and was fixed with self‐tapping screws onto the occiput, followed by cervical screws after reduction. Allograft was placed over the prepared graft bed around the rod‐screw construct on exposed lamina and lateral masses at all fusion levels. A postoperative cervical collar was advised for 8 weeks.

All patients received standard 18‐month anti‐TB chemotherapy. Follow‐ups were conducted at 1, 3, 6, 9, 12, 18, and 24 months postintervention, including clinical, radiographic, and laboratory evaluations. Neck pain severity (VAS‐neck) and complications (e.g., pin‐site infections, hardware failure) were systematically recorded. Radiographic stability was assessed via dynamic cervical X‐rays and CT scans (at 6, 12, and 24 months) to evaluate bone repair and fusion status. Inflammatory markers (erythrocyte sedimentation rate [ESR], C‐reactive protein [CRP]) and TB activity were monitored periodically. Data were prospectively archived in a standardized research database.

### 2.4 Evaluation

Pre‐ and postoperative radiological parameters were systematically evaluated using X‐ray, CT, and MRI. Assessments included osteosclerotic changes and increased bone density in bone destruction areas, resolution or decreased density of necrotic bone (sequestrum), reduction of T2‐weighted hyperintensity in bone marrow and adjacent soft tissues, and abscess volume regression or complete absorption.

The visual analog scale (VAS‐neck), characterized by simplicity and intuitiveness, is a popular tool for measuring pain [25]. Additionally, the neck disability index (NDI) is a common functional status measure with high reliability and internal consistency [26, 27]. We used two quantitative measurements, VAS‐neck and NDI, to assess clinical outcomes. All patients rated their pain intensity on a VAS‐neck and recorded NDI, preoperatively and during follow‐up. Clinical parameters were evaluated and recorded.

ESR is generally elevated many folds in most patients with spinal TB. ESR declines to normal or near‐normal when the active tuberculous lesion is controlled [28]. CRP is an acute inflammatory protein and a component of the innate immune response [29]. As an immune‐based biomarker, it has also been proposed to monitor TB treatment response [30]. Serum or whole‐blood CRP concentrations are widely used in routine clinical practice. ESR and CRP, as markers of infection, were tested and recorded as clinical parameters.

Surgery‐related complications were recorded until the final follow‐up.

### 2.5 Instrument Removal

All patients must meet the following criteria for device removal: (i) radiographic confirmation of bone healing (as in the previously mentioned criteria); (ii) good stability of the neck without localized pressure and longitudinal percussion pains; (iii) no symptoms of TB toxicity, fever, night sweats, malaise, etc.; (iv) relief of preoperative pain symptoms, VAS less than 3, NDI less than 20; (v) Infection indicators (blood sedimentation, CRP, etc.) are normal; (vi) after 6 months of treatment in group H; after 24 months of treatment in group O.

### 2.6 Statistical Analysis

SPSS 22.0 (IBM Corp., Armonk, NY, USA) was used for statistical analysis. Measurement data were tested for normality before further analysis. Continuous variables (e.g., age, BMI, VAS, NDI) were analyzed using the Student's *t*‐test (parametric data), while non‐normally distributed or categorical variables (e.g., sex, disease course categories, segment groups) were compared with the Mann–Whitney *U* test (nonparametric data). Variance of categorical variables was statistically compared using the chi‐square test. A *p*‐value of < 0.05 was considered statistically significant.

## 3 Results

### 3.1 Baseline Data

The baseline clinical characteristics of the study participants are detailed in Table 1. Demographic data were similar between the groups (*p* > 0.05). This suggests that the baseline data for the two patient groups are comparable.

**TABLE 1:** Baseline characteristics of the included patients.

Characteristic	Group H (*n* = 22)	Group O (*n* = 21)	Statistics	*p*
Age (years)^a^	40.6 ± 20.80	38.1 ± 18.48	*t* = 0.38	0.71
Male/female^b^	15/7	13/8	*χ* ^2^ = 0.01	0.92
BMI (kg/m2)^a^	21.3 ± 2.18	22.0 ± 2.58	*t* = 0.95	0.35
Disease course, *n* (%)^b^			*χ* ^2^ = 0.61	0.43
Stage 2	15 (68%)	12 (57%)		
Stage 3	7 (32%)	9 (43%)		
VAS^a^	7.4 ± 1.10	7.2 ± 1.39	*t* = 0.46	0.65
NDI (%)^a^	53.6 ± 10.23	51.4 ± 10.71	*t* = 0.60	0.55
ESR (mm/h)^a^	59.8 ± 22.45	48.7 ± 22.16	*t* = 1.64	0.11
CRP (mg/L)^a^	45.2 ± 25.55	53.2 ± 33.27	*t* = 0.88	0.38
Segment, *n* (%)^b^			*χ* ^2^ = 1.15	0.56
C1‐2	9 (41%)	12 (57%)		
C0‐1	9 (41%)	6 (29%)		
C0‐2	4 (18%)	3 (14%)		
Clinical manifestation				
Night sweats, *n* (%)^b^			*χ* ^2^ = 0.20	0.65
Yes	14 (64%)	12 (57%)		
No	8 (36%)	9 (43%)		
Fatigue, *n* (%)^b^			*χ* ^2^ = 0.43	0.51
Yes	10 (45%)	12 (57%)		
No	12 (55%)	9 (43%)		
Neck movement limitation, *n* (%)^b^			*χ* ^2^ = 0.75	0.39
Yes	18 (82%)	19 (90%)		
No	4 (18%)	2 (10%)		
Fever, *n* (%)^b^			*χ* ^2^ = 0.80	0.37
Yes	14 (64%)	16 (76%)		
No	8 (36%)	5 (24%)		

Abbreviations: BMI, body mass index; CRP, C‐reactive protein; ESR, erythrocyte sedimentation rate; NDI, neck disability index; VAS‐neck, visual analog scale.

^a^ Data are presented as given as mean ± standard deviation.

^b^ Data are presented as the number of patients.

### 3.2 Surgical Assessment

**FIGURE 1:**
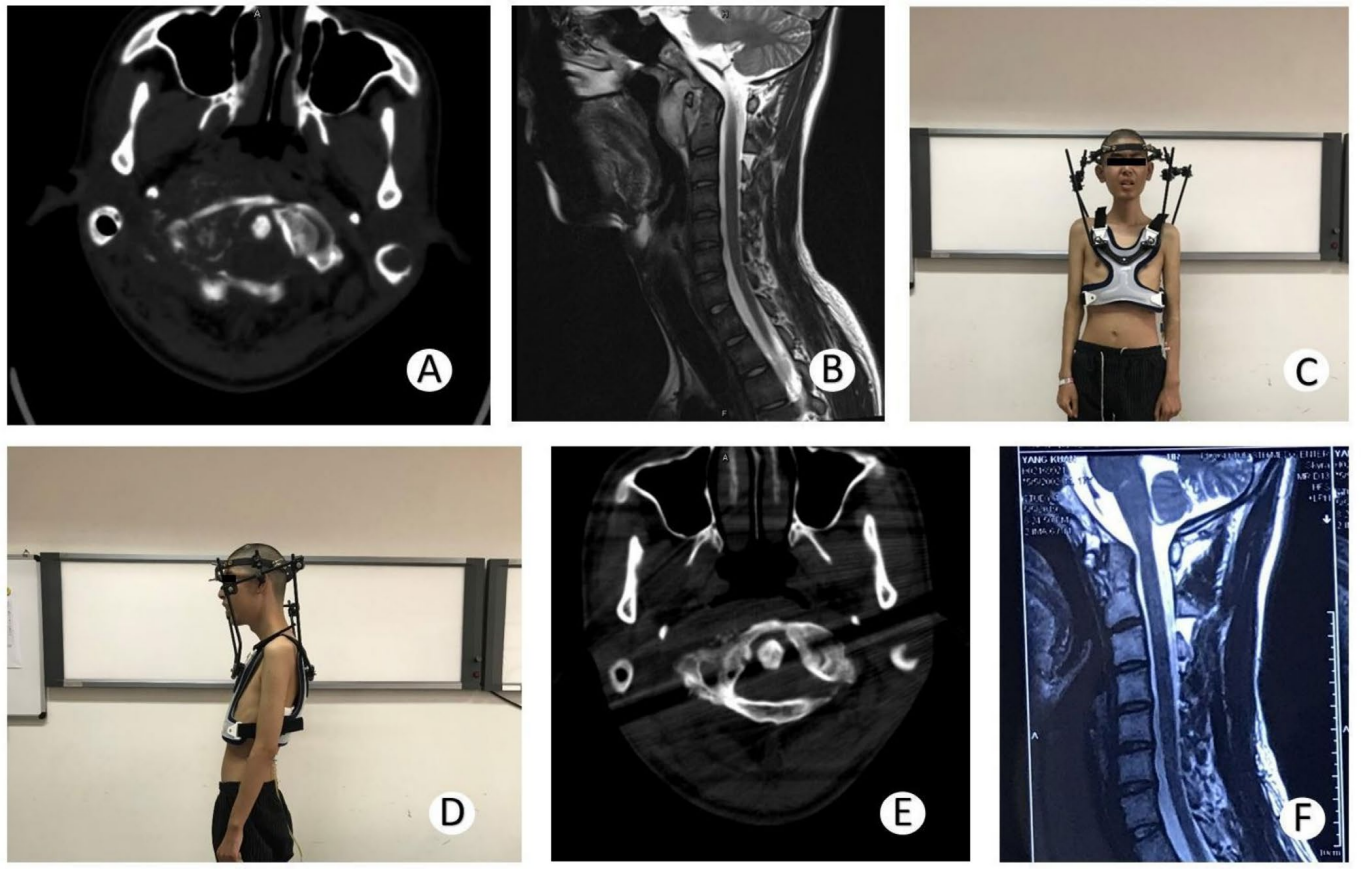
A 16‐year‐old male patient with C0‐1 tuberculosis in Group H. (A) CT axial view showing the destruction of the vertebral body. (B) T2‐weighted MRI showing the retropharyngeal abscess. (C) Front view of the treatment with HV. The patient was treated with oral anti‐tuberculosis drugs and HV immobilization. (D) HV in side view. (E) CT axial view at 9 months of follow‐up. Fewer tuberculosis lesions and bone destruction than before. (F) T2‐weighted MRI at 12 months of follow‐up. Tuberculosis lesions were cured, and no retropharyngeal abscess was observed.

All patients' surgeries were completed successfully. The mean procedural duration was 40 min (range, 30–60 min) for Group H (halo‐vest immobilization) and 235 min (range, 180–300 min) for Group O (OCF). The 22 patients in Group H wore the treatment fixture for an average of 8.2 months (range, 7–11 months) (Figure 1).

**FIGURE 2:**
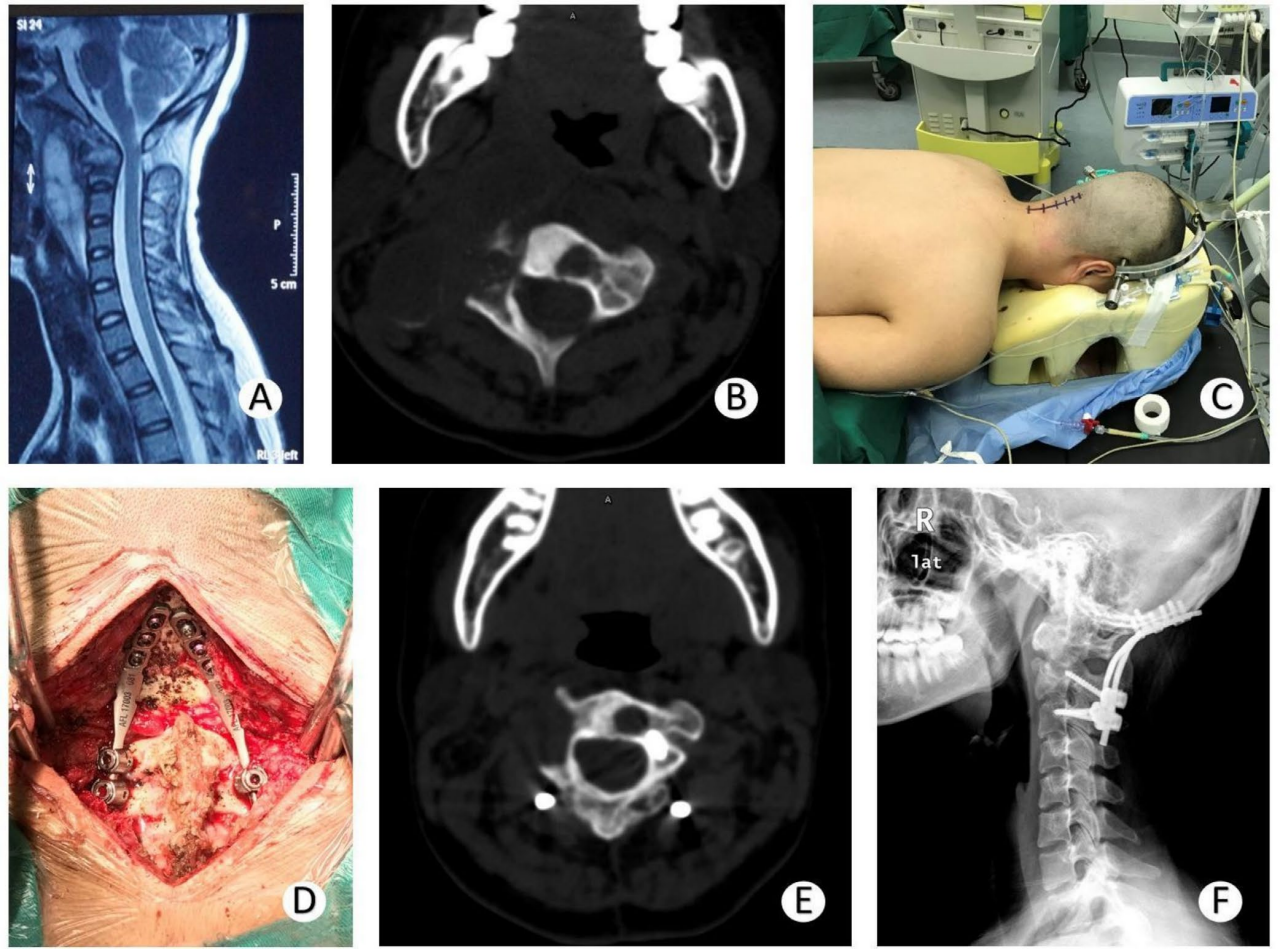
A 14‐year‐old female patient with C1‐2 tuberculosis in Group O. (A) T2‐weighted MRI showing retropharyngeal abscess and abnormal imaging of the odontoid process. (B) CT axial view showing abscess formation and vertebral body destruction cavities. (C) In the prone position, the patient was prepared for surgery. She was treated with OCF and anti‐tuberculosis drugs. (D) The surgical procedure. The plate on the occiput was fixed with cervical screws. (E) CT axial view at 6 months of follow‐up. The abscess was absorbed; the destruction cavity of the vertebral was limited. (F) Lateral X‐ray at 12 months of follow‐up showing occipitocervical fusion fixation. The prevertebral space was normal. The space between the odontoid process and anterior arch of the atlas was normal.

The average intraoperative blood loss for 21 patients in Group O was 223.8 mL (range: 150–450 mL). These patients removed the internal fixation two years later (Figure 2).

Neither group experienced aggravated neurological damage after surgery. TB lesions in all patients healed well during the 12‐month follow‐up period, as shown by radiological examination (Figures 1E,F and 2E,F). After removing the brace or internal fixation, patients in Group H showed greater improvement in cervical flexion‐extension and rotation function than those in Group O.

### 3.3 VAS‐Neck

**FIGURE 3:**
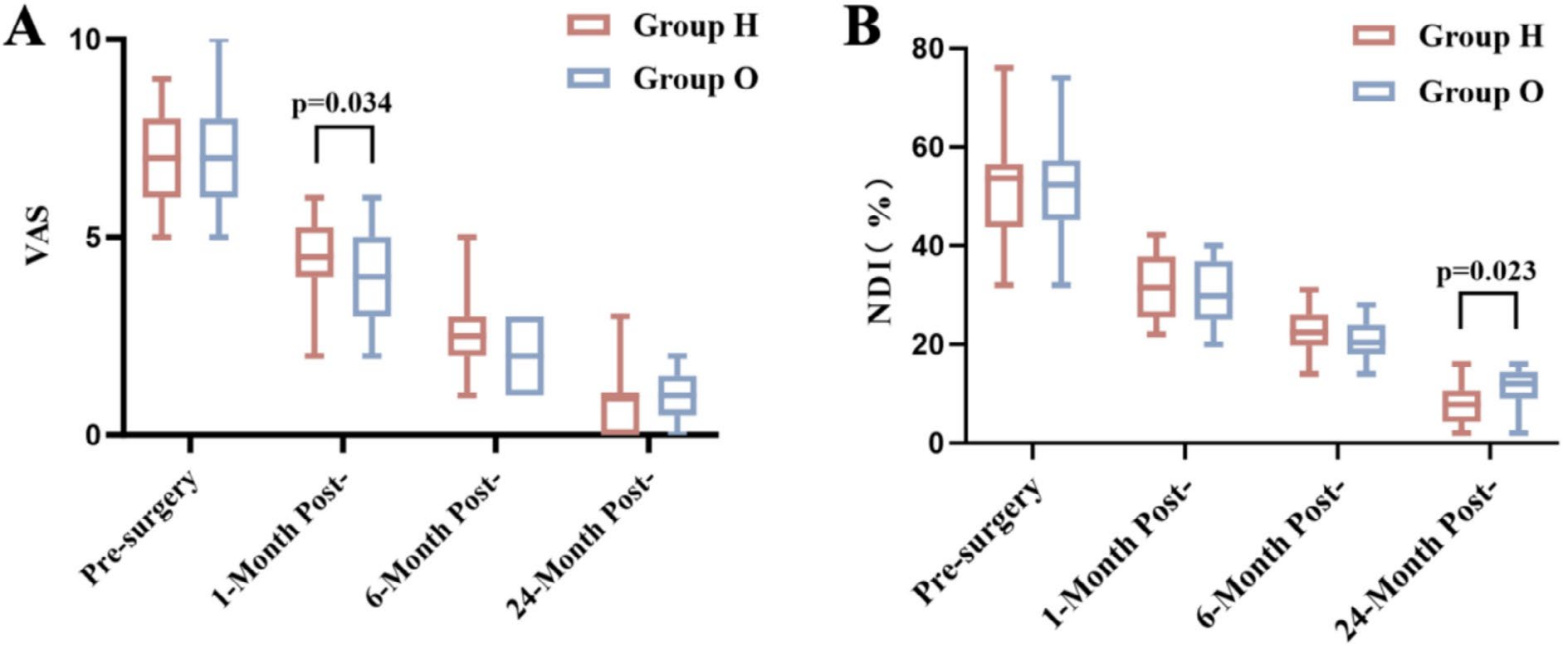
Patients' mean VAS‐neck (A) and NDI (B) scores at the time of pre‐surgery and follow‐up.

After surgery, all patients experienced pain relief. Compared with pre‐surgery scores (Group H = 7.4 ± 1.10, Group O = 7.2 ± 1.39), the VAS scores in both groups showed a statistically significant decrease at each time point (*p* < 0.01) (Figure 3A).

At the 1‐month follow‐up, the VAS‐neck score decreased to 4.7 (±1.14) in Group H and 3.8 (±1.10) in Group O. The difference between the two groups was statistically significant (*p* < 0.05).

At the 6‐month follow‐up, the VAS‐neck score was 2.3 (±0.97) in Group H and 2.1 (±0.85) in Group O. There was no statistically significant difference between the groups (*p* > 0.05).

At the 24‐month follow‐up, the VAS‐neck score was 0.7 (±0.90) in Group H and 0.9 (±0.72) in Group O. There was no statistically significant difference between the groups (*p* > 0.05).

### 3.4 NDI

Compared with pre‐surgery scores (Group H = 53.6 ± 10.23%, Group O = 51.4 ± 10.71%), the NDI scores in both groups showed a statistically significant decrease at each time point (*p* < 0.01) (Figure 3B).

At the 1‐month follow‐up, the NDI score was 31.4 (±6.02) % in Group H and 30.5 (±6.73) % in Group O. There was no statistically significant difference between the groups (*p* > 0.05).

At the 6‐month follow‐up, the NDI score was 22.8 (±4.45) % in Group H and 21.1 (±4.39) % in Group O. There was no statistically significant difference between the groups (*p* > 0.05).

At the 24‐month follow‐up, after the removal of the brace or internal fixation, neck function in Group H was better than in Group O. The NDI score decreased to 7.9 (±4.48) % in Group H and 11.3 (±3.85) % in Group O. The difference between the groups was statistically significant (*p* < 0.05).

## 4 Complications

### 4.1 Group H

Pin‐site infections: Observed in 54.5% (12/22) of patients, presenting as redness, swelling, and exudation at pin insertion sites persisting for 5–7 days. These were managed conservatively with alcohol‐based dressing changes; two cases requiring pin repositioning achieved complete resolution.

Pin loosening: Occurred in 31.8% (7/22) patients, manifesting as reduced fixation stability. Prophylactic daily inspections enabled early detection, with all cases corrected through frame adjustments within 24 h.

Notably, no restrictive ventilation impairment or intracranial pin penetration complications were observed during the treatment course, as confirmed by serial radiographic monitoring.

### 4.2 Group O

Surgical site infections: Two patients (9.5%) developed delayed wound healing characterized by redness, swelling, exudation, and dehiscence, which were successfully managed with secondary suturing within 3–5 days.

Postoperative axial symptoms: 23.8% (5/21) of patients reported persistent cervicothoracic pain, managed through structured rehabilitation exercises and oral nonsteroidal anti‐inflammatory drugs (NSAIDs), with symptom resolution achieved within 1–2 months.

Implant loosening: One patient (4.8%) exhibited minor screw displacement on 12‐month follow‐up radiographs, potentially associated with osteoporosis or excessive cervical motion. This was addressed through anti‐osteoporosis therapy (e.g., bisphosphonates) and activity restriction, demonstrating no progression at the final follow‐up.

Although no direct complications were observed in the present study, OCF for atlantoaxial TB carries substantial risks of severe intraoperative and postoperative adverse events [22]. Intraoperatively, the complex anatomical architecture of the craniocervical junction predisposes to vertebral artery injury with life‐threatening hemorrhage or inadvertent dural tear, increasing the likelihood of postoperative cerebrospinal fluid leakage and secondary infections. Postoperatively, epidural hematoma formation may compress the brainstem or spinal cord, resulting in irreversible neurological deficits or fatal outcomes. Furthermore, in cases with active tuberculous infection, persistent cerebrospinal fluid leakage could facilitate bacterial dissemination, potentially leading to rare but severe tuberculous meningitis [31, 32]. In contrast, noninvasive HV orthosis avoids these surgically induced risks and may serve as a preferable alternative for high‐risk patients requiring stabilization.

### 4.3 ESR and CRP

**FIGURE 4:**
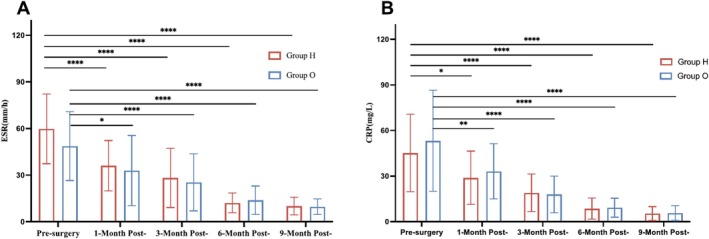
Patients' mean ESR (A) and CRP (B) values at the time of pre‐surgery and follow‐up. Data are presented as mean ± s.d. Statistical analysis was conducted using two‐way ANOVA with multiple comparisons using Tukey's post‐test. *p < 0.05, **p < 0.01, ***p < 0.001, ****p < 0.0001.

Compared with pre‐surgery values (ESR: Group H = 59.8 ± 22.45 mm/h, Group O = 48.7 ± 22.16 mm/h; CRP: Group H = 45.2 ± 25.55 mg/L, Group O = 53.2 ± 33.27 mg/L), the ESR and CRP values in both groups showed a statistically significant decrease at each time point (*p* < 0.01) (Figure 4).

At the 1‐, 3‐, 6‐, and 9‐month follow‐ups, the ESR values (mm/h) in Group H were 37.3 (±15.99), 29.4 (±19.17), 12.5 (±6.92), and 10.7 (±5.97); the values in Group O were 32.6 (±24.71), 26.5 (±19.47), 14.4 (±10.09), and 10.1 (±5.26), respectively. At the same time points, the CRP values (mg/L) were 30.7 (±18.50), 18.9 (±13.47), 9.4 (±7.32), and 5.8 (±4.77) in Group H, and 34.3 (±19.86), 17.9 (±12.01), 10.3 (±6.41), and 5.9 (±5.44) in Group O, respectively. There was no statistically significant difference in ESR and CRP values between the groups at any time point (*p* > 0.05). Figure 4. Patients' mean ESR (A) and CRP (B) values at the time of pre‐surgery and follow‐up.

## 5 Discussion

### 5.1 Principal Findings

This study demonstrates that halo‐vest immobilization serves as an effective and minimally invasive therapeutic strategy for CVJ TB. It significantly alleviates pain, restores functional capacity, and achieves durable inflammatory control while maintaining biomechanical stability. Compared with surgical interventions, this approach circumvents the risks inherent to open surgical interventions, notably minimizing procedural trauma, reducing healthcare expenditures, and obviating perioperative complications; it is particularly advantageous for patients with medical comorbidities or intricate anatomical pathologies that contraindicate conventional surgical management. Although transient complications such as pin‐site issues were observed, all were manageable through standardized protocols without long‐term sequelae. The findings underscore its clinical utility as a viable non‐surgical alternative, especially for early‐stage disease or cases requiring conservative management.

### 5.2 Comparison With Existing Evidence

After the diagnosis of spinal TB, primary therapy involves immobilization and antituberculous chemotherapy. The CVJ complex houses essential neural and vascular structures, while also being the most mobile segment of the spine [33]. The superior articular surfaces of the atlas interface with the occipital condyles in the O‐C1 joints, primarily facilitating flexion‐extension movements [34]. Since the atlas communicates inferiorly with the axis through flat, wide articular facets [35, 36], the C1‐2 joints primarily facilitate rotational movement. Treating spinal TB is challenging due to the necessity of immobilization. In 1974, Tuli reported a large series on CVJ TB and proposed prolonged bed rest and skull traction for treating these patients [37, 38]. Since then, the treatment for patients with CVJ TB has continued to evolve. Some experts recommend surgical intervention for all patients [15, 19, 39]. Sanjiv Sinha suggested that surgery is beneficial for early patient mobilization. Open surgical procedures can obtain biopsy tissue and perform radical excision of epidural granulation tissue/abscess and infected bone using microsurgical techniques [40].

However, compared with conservative treatment, surgery is associated with higher costs and a higher complication rate. Furthermore, the cervical blood supply is abundant [41], and conservative treatment for cervical TB is more effective compared with treatment for other forms of spinal TB. Advocates of non‐surgical treatment have demonstrated equally favorable clinical outcomes [14, 17, 42]. Kumar A et al. reported a case of rotatory atlantoaxial subluxation due to CVJ TB, successfully treated with a Philadelphia collar [17]. Similarly, after reviewing 26 cases of CVJ TB, Arora S et al. advocated that treatment with a HV is a safe and reliable method for managing CVJ TB [43]. This finding is consistent with the results of our study. In CVJ TB cases without spinal cord compression, lesion removal is unnecessary; immobilization combined with anti‐TB drugs can achieve satisfactory outcomes. As a conservative treatment, a HV restricts motion in the upper cervical spine more effectively than other orthoses [44]. Considering that HV treatment results in less blood loss, reduced surgical pain and cost, shorter hospital stays, and fixed treatment duration, it offers more advantages.

In our study, neither group experienced severe complications. Some patients in Group H experienced varying degrees of pin tract inflammation during follow‐up. Presumably, the pathogens originated from opportunistic bacteria colonizing the scalp. Inflammation at the pin tracks cannot be entirely avoided due to the invasive and external nature of the HV brace. Additionally, there were micro‐movements in the fixation pins during use. Fortunately, inflammation was confined to the region around the pin tracks, facilitated by the abundant blood supply of the scalp. Additionally, some patients in Group H had loose fixation pins. Patients were instructed to promptly return to the hospital for adjustments if the pins became loose to maintain brace firmness and treatment efficacy. Two patients in Group O required readmission and secondary suturing. Although Group O did not experience complications such as injury to key neurological and vascular structures, these complications can be potentially fatal.

According to the VAS‐neck results, patients in Group O experienced statistically significant pain relief compared with Group H at the 1‐month follow‐up, but the differences in the mid‐ to long‐term were not statistically significant. This is likely due to TB lesions not being fully healed in the early stages of treatment. Dual immobilization with internal fixation and a cervical collar in Group O provided greater stability than HV treatment. However, at end‐stage follow‐up, we observed that pain relief outcomes were not significantly different between the two groups, indicating that short‐term differences did not decisively affect the healing of TB lesions. The NDI scores indicated no significant difference between the two groups before removal of the fixation devices. After removal of the fixation device, Group H showed better improvement in neck function compared with Group O at the final follow‐up. Due to fusion of the affected segment and scarring of soft tissue from the operation, Group O had a lower range of motion compared with Group H. Additionally, the cervical spine can experience slight movement during HV immobilization, leading to better neck function compared with patients who underwent open surgery. ESR and CRP values in all patients significantly decreased compared with pre‐surgery levels, with no statistical difference observed between the two groups at any time point. This indicates that both treatments have similar effectiveness in healing TB lesions. Lesion recovery primarily results from bacteria elimination by anti‐TB drugs, with immobilization playing a supportive role. We believe that despite HV being less stable than operative fixation, it can effectively complement anti‐TB treatment.

### 5.3 Strengths and Limitations

The HV orthosis offers a minimally invasive alternative for surgically managing CVJ TB, providing triplanar stabilization to ensure biomechanical rigidity and mitigate the risk of secondary neurological compromise, while circumventing open surgical risks such as iatrogenic vascular injury. By effectively immobilizing cervical motion, this modality facilitates inflammatory resolution through mechanical load redistribution, demonstrating particular efficacy in early‐stage disease (Geol stage 2). However, its efficacy is constrained in advanced instability (Geol Stage 3), necessitating surgical stabilization. Key limitations include prolonged immobilization duration (typically 6–9 months), which challenges patient compliance, and the requirement for rigorous multidisciplinary management—encompassing anti‐TB chemotherapy, pin‐site care, and serial radiographic monitoring—to mitigate transient complications (e.g., pin‐site infections). Thus, while HV provides a balanced approach for select cases, its success depends on meticulous patient selection and protocol adherence.

### 5.4 Clinical Implications and Future Directions

HV immobilization offers a safe, non‐surgical approach for CVJ TB, particularly in early‐stage disease (Geol Stage 2) and high‐risk patients. By stabilizing the CVJ to promote inflammatory resolution while avoiding surgical risks, HV serves as an evidence‐based conservative option, especially in resource‐limited settings. Future studies should define HV's adjunctive role in advanced instability (Stage 3), develop lightweight devices to minimize complications, and explore synergies with biologic agents. Long‐term data are needed to assess fusion durability and recurrence risks, refining clinical guidelines.

## 6 Conclusion

During the 24‐month follow‐up of patients with CVJ TB, no statistically significant difference was observed in the resolution of TB lesions between the two treatment options. Patients treated with HV showed greater improvement in neck function compared with those treated with OCF. Given the high cost and risks associated with open surgery, we advocate conservative treatment with HV as a reliable and effective option for these patients.

## Author Contributions

All authors had full access to the data in the study and took responsibility for the integrity of the data and the accuracy of the data analysis. Conceptualization: Yunfeng Wu and Xiyou Yang. Methodology: Yunfeng Wu and Xiyou Yang. Investigation: Yunfeng Wu and Long Yu. Formal analysis: Ning Liu and Shangjie Yang. Resources: Long Yu and Ning Liu. Writing – original draft: Yunfeng Wu and Xiyou Yang. Writing – review and editing: Xu Cui. Visualization: Xu Cui. Supervision: Xu Cui. Funding acquisition: Xu Cui.

## Conflicts of Interest

The authors declare no conflicts of interest.

## Data Availability

Anonymized participant data for this study will be available upon request to the corresponding author.
